# Status epilepticus and the presence of SARS‐COV‐2 in the cerebrospinal fluid: A case report

**DOI:** 10.1002/ccr3.6214

**Published:** 2022-08-09

**Authors:** Sara Abdolahi, Rezan Ashayeri Ahmadabad, Ali Gorji, Zahra Mirzaasgari

**Affiliations:** ^1^ Shefa Neuroscience Research Center Khatam Alanbia Hospital Tehran Iran; ^2^ Neuroscience Research Center Mashhad University of Medical Sciences Mashhad Iran; ^3^ Department of Neurosurgery and Neurology Westfälische Wilhelms‐Universitat Münster Münster Germany; ^4^ Epilepsy Research Center, Department of Neurosurgery Westfälische Wilhelms‐Universitat Münster Münster Germany; ^5^ Department of Neurology, Firoozgar hospital, School of Medicine Iran University of Medical Science Tehran Iran

**Keywords:** COVID‐19 infection, neuroinvasive, seizure

## Abstract

A growing number of studies indicate a broad range of neurological manifestations, including seizures, occur in patients with COVID‐19 infection. We report a 29‐year‐old female patient with status epilepticus and positive SARS‐CoV‐2 in the cerebrospinal fluid. Our findings support previous reports suggesting seizure as a possible symptom of COVID‐19 infection.

## Graphical abstract

The severe acute respiratory syndrome coronavirus‐2 (SARS‐CoV‐2) can directly target the central nervous system (CNS). Our findings suggest that neurologic symptoms such as seizures may be the initial presentation of COVID‐19. Therefore, In patients presenting with seizures and/or status epilepticus, to consider SARS‐Cov‐2 infection and cerebrospinal fluid (CSF) analysis is advised.

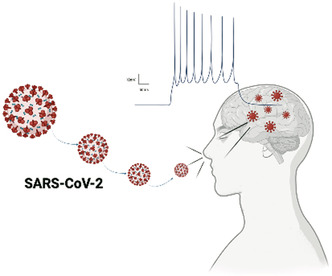

## 1 INTRODUCTION

Severe acute respiratory syndrome coronavirus‐2 (SARS‐CoV‐2) could infect various organs by different mechanisms. This includes the central nervous system (CNS) and peripheral nervous system.^2^ Examination of autopsy tissue from the patients with SARS‐CoV‐2 infection demonstrated that this virus has the potential to enter the CNS via attacking the vascular system and damaging the blood–brain barrier.^3^ Furthermore, post‐mortem examination of patients infected with COVID‐19 has revealed that SARS‐CoV‐2 could directly target cortical neurons, which may be associated with immune cell infiltration in the brain.^4^, ^5^ Among various neurological manifestations, the occurrence of seizures during the acute phase of SARS‐CoV‐2 infection has been reported.^5^, ^6^ Here, we report a patient with status epilepticus and a positive SARS‐CoV‐2 ribonucleic acid test of the cerebrospinal fluid (CSF).

## 2 CASE PRESENTATION

**FIGURE 1:**
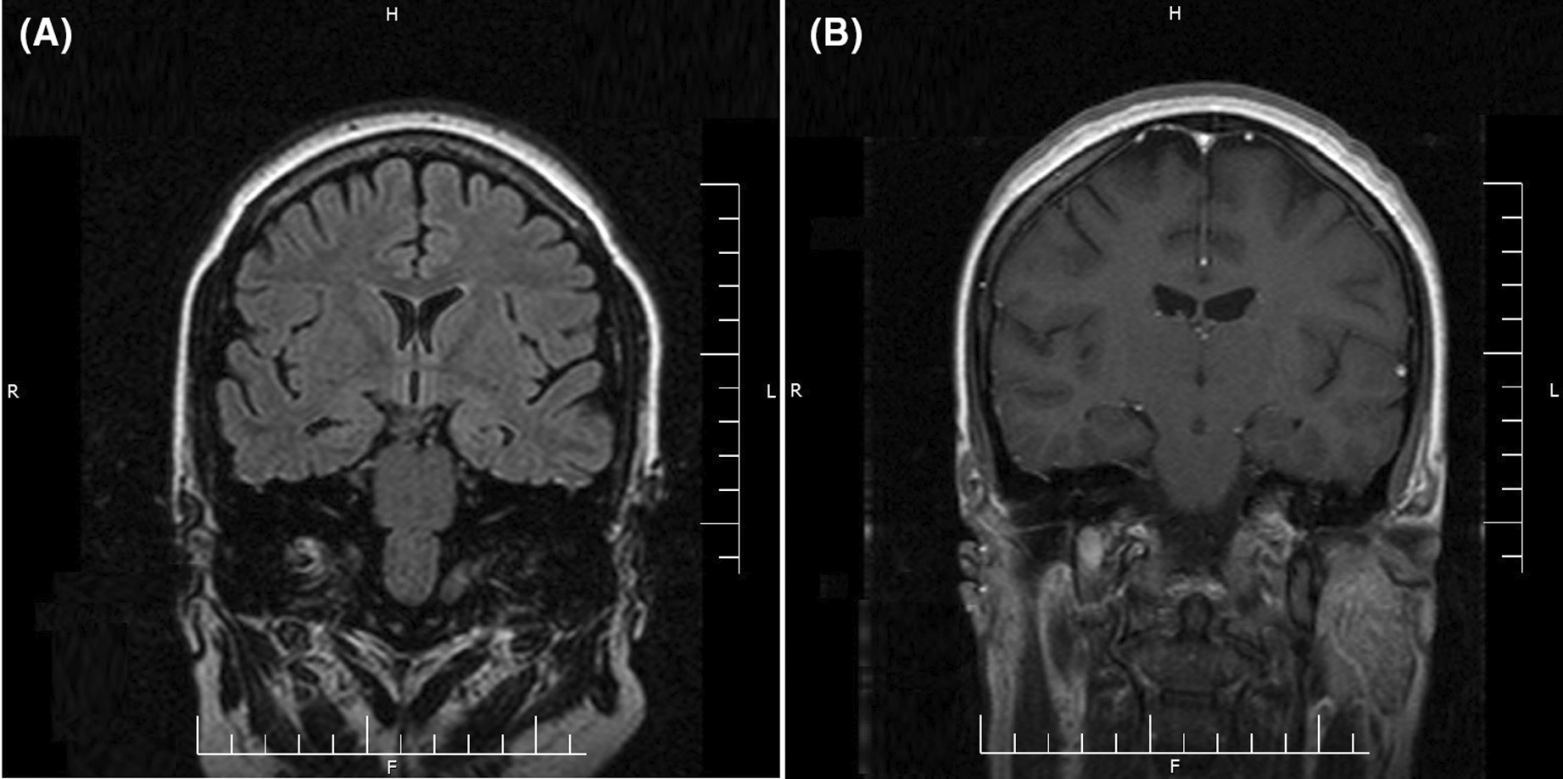
Coronal T2‐weighted Flair non‐contrast brain magnetic resonance imaging (MRI) (A) and coronal T1‐weighted contrast‐enhanced brain MRI (B)

**FIGURE 2:**
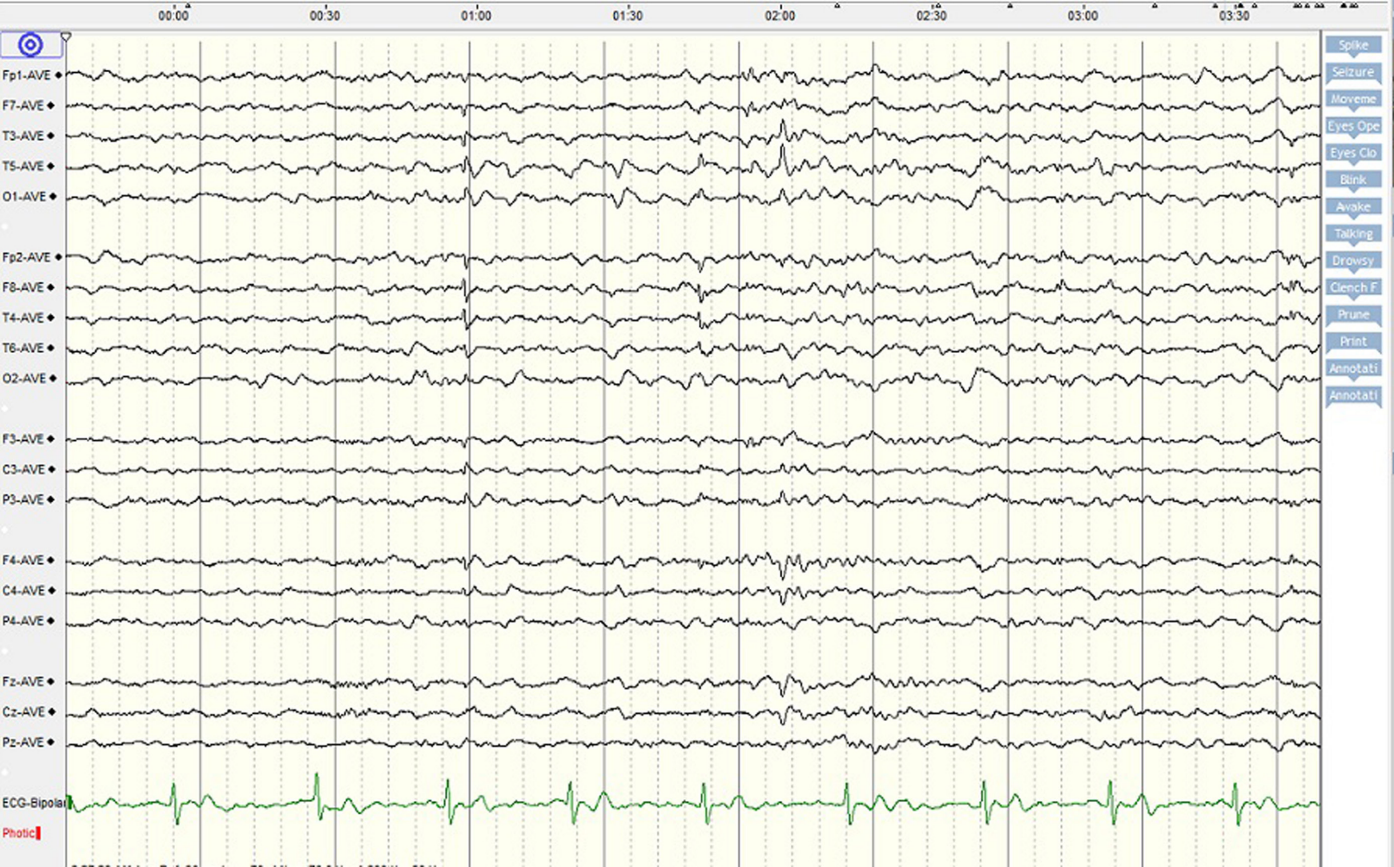
EEG showed bilateral temporal epileptiform discharges

A 29‐year‐old female patient was brought to the emergency department with frequent episodes of generalized tonic–clonic seizures. There was no history of prior seizure, headache, behavioral change, hemiparesis, and any other neurological symptoms. Likewise, she had no other symptoms regarding involvement of other organs by COVID‐19 at the time of admission. The seizures were stopped with a loading dose of levetiracetam (3000 mg per day). Initial brain and chest computed tomography scans as well as brain magnetic resonance imaging with and without contrast did not show any abnormality (Figure 1). Using the real‐time polymerase chain reaction (RT‐PCR) test, the nasopharyngeal swab sample was positive for SARS‐CoV‐2. CSF analysis indicated normal opening pressure with normal glucose and protein levels. The CSF culture was negative for different microorganisms. Antibodies responsible for autoimmune encephalitis were also negative. However, the RT‐PCR test for SARS‐CoV‐2 in the CSF was positive. The electroencephalogram revealed epileptiform discharges in bilateral temporal areas (Figure 2). The summary of the laboratory data is shown in Table 1.

**TABLE 1:** Laboratory data of the patient

Variable		Results	Reference value
Blood test	WBC	1**6**.9 × 1000/mm^3^	4.0–10
	RBC	5.39 × 10^6^/mm^3^	F:4.2–5.4
	Hb	9.4 (g/dl)	F:12–16
	MCV	59.2 fl	77–97
	Plt	419 × 10^3^/mm^3^	140–440
	Ferritin	14.5 ng/ml	12–150 ng/ml
	AST	23 U/L	<31
	ALT	16 U/L	<31
	ALP	142 IU/L	Up to 270
	CPK	88 U/L	26–140
	CK‐MB	10 U/L	Up to 24
	D‐Dimer	0.5 μg/ml	0–0.6
	ESR	22 mm/h	Female: <20
	CRP Quantitative	3 mg/L	Normal: Up to 6
	Ca	10 mg/dl	8.6–10.3
	Mg	1.9 mg/dl	Adult: 1.8–2.6
	Na	140 mmol/L	135–145
	K	4 mmol/L	3.5–5.5
	Creatinine	1.1 mg/dl	Female: 0.6–1.3
	LDH	325 U/ml	225–500
	Total protein	7.1 g/dl	Adult: 6.6–8.8
	Blood Sugar	112 mg/dl	100–145
CSF	WBC	0	0–5
	RBC	10 cell	0–6
	Glucose	79 mg/dl	50–80
	Total Protein	15 mg/dl	15–45
	*LDH*	34 U/L	0–40
	CSF culture:	NO growth	–
PCR	Nasopharynx for COVID‐19 PCR:	Positive	Detected: positive Non‐detected: negative
	CSF for COVID‐19 PCR:	Positive	Detected: positive Non‐detected: negative

Abbreviations: ALP, alkaline phosphatase; AST, aspartate aminotransferase; Ca, calcium; CK‐MB, creatine kinase‐MB; CPK, creatine phosphokinase; CRP, C‐Reactive protein; *ESR*, erythrocyte *sedimentation rate*; Hb, hemoglobin; K, potassium; LDH, lactate dehydrogenase; MCV, mean corpuscular volume; Mg, magnesium; Na, sodium; Plt, platelet; RBCs, red blood cells; WBCs, White blood cells.

During hospitalization, the patient received a maintenance dose of levetiracetam (3000 mg/day) and sodium valproate (1600 mg/day). The antiviral treatment was also started with 200 mg remdesivir on Day 1, followed by 100 mg daily for 4 days. After one week, the patient was discharged from the hospital with no seizure. The patient remained seizure‐free during 2 months' follow‐up.

## 3 DISCUSSION

Various investigations have reported different neurological disorders attributable to COVID‐19, including cerebrovascular accidents, transverse myelitis, Guillain–Barre syndrome, and encephalitis.^7^, ^8^ As other possible diseases were excluded through suitable clinical and laboratory tests, this report suggests the neuroinvasive potential of SARS‐CoV‐2.

Increasing evidence suggests that SARS‐CoV‐2 might have neuro‐invading potential, which may cause clinical symptoms and brain damage.^9^ It has been demonstrated that other coronaviruses may invade the CNS implication and cause an influx of inflammatory cytokines. Human coronavirus OC43 *(*HCoV*‐*OC43) is capable of invading neural cells in vitro and causing widespread neuronal damage.^10^ Infection with other coronaviruses, such as SARS and the Middle East Respiratory Syndrome viruses, was associated with various neurological manifestations.^11^ It has been reported that SARS‐CoV‐1 in mice transgenic for the human angiotensin‐converting enzyme‐2 (ACE2) receptor can attack the CNS.^12^ Like SARS‐CoV‐1, SARS‐CoV‐2 uses the ACE2 receptor to infect the human cells. Therefore, SARS‐CoV‐2 may target the CNS through this receptor.^13^ In summary, this report supports the possibility of seizures in patients with COVID‐19 infection in the absence of respiratory symptoms. More investigations are needed to determine the potential mechanisms that lead to seizures following SARS*‐*CoV*‐*2 infection. In patients presenting with seizures and/or status epilepticus, to consider SARS‐CoV‐2 infection and CSF analysis is advised.

## AUTHOR CONTRIBUTIONS

All authors have read and approved the manuscript. SA drafted, did background research, reviewed results, and revised the manuscript. RA reviewed results and revised the manuscript. AG did background research, reviewed results, and revised the manuscript. ZM involved in patient management, collected history, did background research, reviewed results, and revised the manuscript.

## CONFLICT OF INTEREST

The authors declare no conflict of interest.

## ETHICAL APPROVAL AND CONSENT TO PARTICIPATE

This study was conducted in accordance with the Declaration of Helsinki.

## CONSENT

Written informed consent was obtained from the patient for publication of this Case report and any accompanying images. A copy of the written consent is available for review by the Editor of this journal.

## Data Availability

Not applicable.
